# Label-Free Prediction of Fluorescently Labeled Fibrin Networks

**DOI:** 10.34133/bmr.0211

**Published:** 2025-05-28

**Authors:** Sarah Eldeen, Andres Felipe Guerrero Ramirez, Bora Keresteci, Peter D. Chang, Elliot L. Botvinick

**Affiliations:** ^1^ Department of Mathematical, Computational, and Systems Biology, University of California, Irvine, Irvine, CA, USA.; ^2^ Department of Radiological Sciences and Computer Sciences, University of California, Irvine, Irvine, CA, USA.; ^3^ Department of Biomedical Engineering, University of California, Irvine, Irvine, CA, USA.; ^4^ Beckman Laser Institute and Medical Clinic, University of California, Irvine, Irvine, CA, USA.; ^5^ Edwards Lifesciences Foundation Cardiovascular Innovation and Research Center, University of California, Irvine, Irvine, CA, USA.; ^6^ Department of Surgery, University of California, Irvine, Irvine, CA, USA.

## Abstract

While fluorescent labeling has been the standard for visualizing fibers within fibrillar scaffold models of the extracellular matrix (ECM), the use of fluorescent dyes can compromise cell viability and photobleach prematurely. The intricate fibrillar composition of ECM is crucial for its viscoelastic properties, which regulate intracellular signaling and provide structural support for cells. Naturally derived biomaterials such as fibrin and collagen replicate these fibrillar structures, but longitudinal confocal imaging of fibers using fluorescent dyes may impact cell function and photobleach the sample long before termination of the experiment. An alternative technique is reflection confocal microscopy (RCM) that provides high-resolution images of fibers. However, RCM is sensitive to fiber orientation relative to the optical axis, and consequently, many fibers are not detected. We aim to recover these fibers. Here, we propose a deep learning tool for predicting fluorescently labeled optical sections from unlabeled image stacks. Specifically, our model is conditioned to reproduce fluorescent labeling using RCM images at 3 laser wavelengths and a single laser transmission image. The model is implemented using a fully convolutional image-to-image mapping architecture with a hybrid loss function that includes both low-dimensional statistical and high-dimensional structural components. Upon convergence, the proposed method accurately recovers 3-dimensional fibrous architecture without substantial differences in fiber length or fiber count. However, the predicted fibers were slightly wider than original fluorescent labels (0.213 ± 0.009 μm). The model can be implemented on any commercial laser scanning microscope, providing wide use in the study of ECM biology.

## Introduction

The extracellular matrix (ECM) is a complex meshwork that supports and facilitates cellular communication within tissues, regulating processes such as migration, differentiation, and tissue morphogenesis [1,2]. The natural makeup of the ECM comprises noncellular fibrillar elements, such as collagen, elastin, fibronectin, and several other glycoproteins, which grant it viscoelasticity and nonlinear elastic properties [2,3]. Initially, the ECM was thought to serve merely as a scaffold for maintaining organ and tissue integrity. However, the numerous syndromes caused by ECM mutations emphasize its critical role in biological signaling in vivo [3,4]. There is an association between ECM integrity and diseases, and understanding the role of each ECM component is challenging due to the complexity of the ECM and its dynamic nature [5–8].

Predominantly, studying ECM architecture and associated cellular response is conducted within 3-dimensional (3D) cell culture scaffolds. Such biocompatible scaffolds can be broadly categorized into nanoporous materials, such as alginate and polyethylene glycol (PEG) scaffolds, or natural fibrous biomaterials, such as type 1 collagen and fibrin [9–11]. PEG scaffolds are commonly used in tissue engineering and regenerative medicine due to their tunable physical and chemical properties and versatility in achieving desired nanoporosity, hydrophilicity, binding domains, etc. [12–15]. However, their isotropic, homogeneous microstructure does not recapitulate the fibrillar nature of native ECM, which is crucial for mechanical transduction and force propagation [14,16]. Alternatively, naturally derived fibrillar scaffolds such as collagen and fibrin can mimic ECM mechanical properties and architecture [17].

Typically in fibrillar scaffolds, fiber lengths ranges from 2 to 30 μm [18]. Resolving ECM structure thus requires imaging tools with enhanced optical sectioning capability such as laser scanning confocal microscopy (LSM), based on a developed technique by M. Minsky in 1955 [19]. LSM of fluorescently labeled scaffolds enables visualization of deep fibers (1 to 2 mm) at high resolution in both transverse and longitudinal planes [20]. These diffraction-limited thin transverse sections are used to reconstruct scaffold 3D microstructure within an ECM volume [20]. 3D imaging can identify deformation within the scaffold and track cell interactions in real time during experiments that can last for weeks or longer [21,22]. Unfortunately, such labeled samples become photobleached in a process generating free radicals that can influence cell behaviour through processes such as DNA oxidation [23–25]. Furthermore, exposing cells to high-intensity laser light during LSM can induce DNA damage, especially with lasers of shorter wavelength near the ultraviolet band [26]. An alternate approach to longitudinal LSM is chemical fixation of an ECM followed by fluorescent labeling against target molecules. However, this technique requires terminating samples at each time point (e.g., [26]), precluding longitudinal studies and instead averaging behaviors over time across multiple samples. Such studies necessitate a complex and time-intensive protocol of fixation, labeling, and imaging of each sample.

**Fig. 1.**
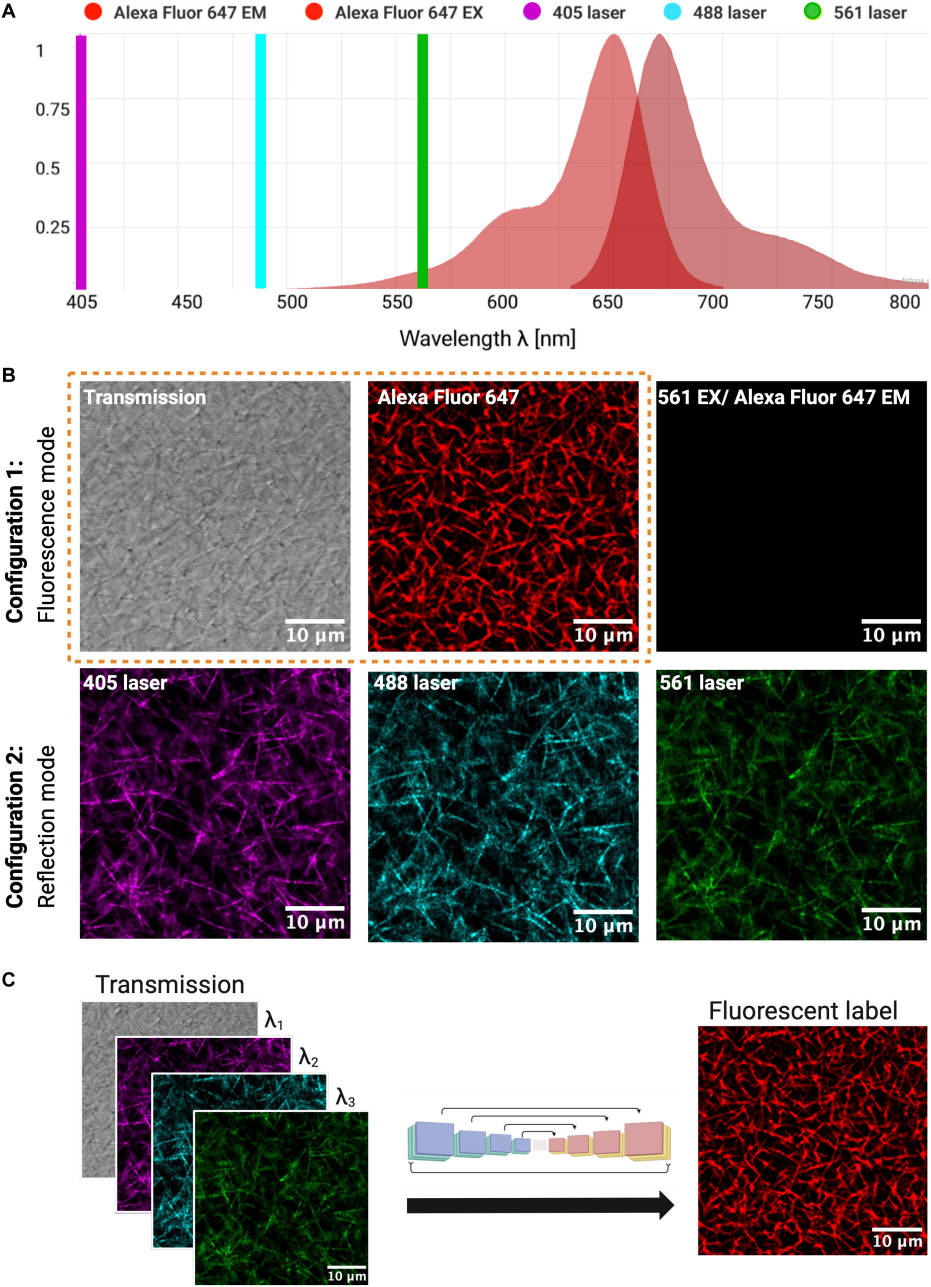
Experimental design. (A) Spectra showing 3 laser emission peaks at 405, 488, and 561 nm, and the excitation/emission spectra of Alexa Fluor 647 nm used to label fibers. Produced by FPBase [45]. (B) Confocal optical sections of a fibrin scaffold acquired in 2 configurations, where a configuration is defined as a unique set of optical filters, dichroic mirrors, and light paths for simultaneously acquiring a set of images. Configuration 1: (bound by orange dashed border) 488-nm laser for transmission, 640-nm laser for exciting Alexa 647 fluorescence, and bandpass filtering to detect Alexa 647 emission. The image in column 3 tests for excitation of Alexa 647 by the 561-nm laser used for RCM. Configuration 2: RCM at 3 laser wavelengths, 405, 488, and 561 nm. (C) Schematic diagram illustrating prediction of fluorescent fibers from label-free images acquired by transmission and RCM.

An alternate modality that can image ECM fibers is reflection confocal microscopy (RCM) that avoids challenges associated with fluorescent labeling of the ECM [27,28]. This method relies on endogenous contrast, utilizing the intrinsic properties of the specimen for imaging, without the need for exogenous labels. RCM is wavelength-dependent, where the choice of illumination wavelength affects the depth, quality, and resolution of images. Further, scattering coefficients are wavelength-dependent and may introduce a level of independence between images at multiple unique laser wavelengths [29]. Therefore, for this study, we labeled fibrin ECMs with the fluorescent dye (Alexa 647). ECMs were then imaged in transmission mode by one laser, and RCM at 3 laser wavelengths (405/488/561 nm), which do not excite the fluorescence. Additionally, the same ECMs were imaged in fluorescence using a 640-nm laser as illustrated in Fig. 1A. As can be seen in Fig. 1B, transmission, fluorescence (Alexa 647), and reflection (405/488/561) images carry different information. The transmission image (top left) reveals detailed structures of the fibrillar sample, but from a thick focal volume, and thus cannot assign the depth of each fiber. In contrast, the RCM images (bottom row: 405/488/561) provide optical sectioning, thus improving depth resolution. However, while RCM detects fibers oriented along the transverse (image) plane, detection fails as orientation has components along the longitudinal axes, and tends to miss important structural features like fiber intersections (nodes) [30]. The fluorescence confocal imaging overcomes these limitations and provides detailed structural information, making it highly advantageous for accurate 3D reconstruction and resolving fine sample details. This observation motivated our method to predict the fluorescently labeled image (Fig. 1C), which we refer to as ground truth (GT).

In this paper, we develop a deep learning approach to virtually stain samples using RCM and transmission images, thereby recovering the missing fibers and gaining structural information about the scaffold (Fig. 1C). Our method extends known frameworks for image-to-image mapping tasks, commonly implemented using fully convolutional architectures such as the U-Net, to learn a mapping function between 2 domains that exploits nonlinear pairwise relationships between the pixels [31]. Recent work in histology has illustrated the use of image-to-image mapping convolutional neural networks (CNNs) to predict fluorescent labels from naive standard nonfluorescent images of histological samples [32,33]. Here, we investigate the use of this technique to virtually label ECM fibers within a 3D fibrin scaffold. The input channels are label-free and exploit the modalities of RCM at 3 laser wavelengths and transmitted light microscopy at a single laser wavelength (Fig. 1A and B), both of which are standard features of any commercial laser scanning confocal microscopes. Predictions are compared to GT using statistical metrics for image reconstruction error as well as derived scaffold metrics such as fiber length, fiber width, and fiber count.

## Materials and Methods

### Scaffold preparation

Bovine stock fibrinogen (MilliporeSigma, F8630-10G) was dissolved in phosphate-buffered saline (PBS; Gibco) and filtered using Thermo Fisher Scientific Nalgene Rapid-Flow Disposable Filter Units (974101). Fibrinogen solution was prepared at 2.5, 5, and 10 mg/ml concentrations and filtered using polyethersulfone 0.22-μm 30-mm-diameter syringe filter (Genesee Scientific 25244) and 10-ml syringe (Henke-Ject 21M29C8). A 1-ml filtered fibrinogen solution was added to 20 μl of bovine thrombin (4 U/ml, Sigma, SLBW2056) and swirled around quickly inside a 35-mm glass bottom dish (MatTek) before polymerization. The swirl method involves tilting the glass dish by hand while moving the fluid in a circular motion to cover the entire dish surface. Each sample was incubated at room temperature for 5 min before being placed in the incubator at 37 °C and 5% CO_2_ for 30 min prior to labeling. Fibers were labeled with Alexa Fluor 647 NHS ester (succinimidyl ester) (Thermo Fisher Scientific A20006) by adding 2 mg/ml of the dye to the sample and incubating it for 15 min on the shaker, followed by 3× washes using PBS.

### Data acquisition

For the training set, five 3D confocal image stacks of 4 different samples (2.5 mg/ml) were acquired with 100-nm step size. Each training set of images was 318.20 × 318.20 × 2 μm^3^, with an image scan area resolution of 4,096 × 4,096 pixels^2^, a pixel size of 77.69 nm, and a pixel dwell time of 4 μs. For the test set, a single 3D image stack was acquired of dimension 40 × 40 × 16 μm^3^ with identical pixel size and image stack step size but with 2-μs pixel dwell time. For each focal plane, 2 sets of confocal images were acquired using an Olympus FV3000 laser scanning confocal microscope and an Olympus 40× silicon oil immersion objective lens (numerical aperture = 1.25). Details of Olympus Fluoview software configuration can be found in Fig. S1. We acquired images in 2 different FV3000 internal optic configurations to accommodate different light paths associated with reflection and fluorescence modalities. One main difference between the 2 configurations is the use of a 10/90 beam splitter for reflection so only 10% of light intensity can reach the sample. Laser lines and dye excitation/emission spectra are shown in Fig. 1A. Briefly for configuration 1, the GT fluorescence and transmission images are acquired simultaneously (Fig. 1B). Note that the 561-nm laser used for RCM does overlap the tail of the excitation spectra of Alexa 647. We tested if any excited fluorescence contributed to the 561-nm RCM image, which would bias our technique. For this one test, we reconfigured FV3000 in a standard fluorescence mode with the dye being excited by the 561-nm laser at the same power used for RCM. No fluorescence was detected (Fig. 1B, 561 EX/Alexa Fluor 647 EM). Configuration 2 acquired all 3 RCM images simultaneously using the 405-, 488-, and 561-nm lasers (refer to Fig. 1B, Configuration 2 for an example).

### Data preprocessing

#### Normalization

**Fig. 2.**
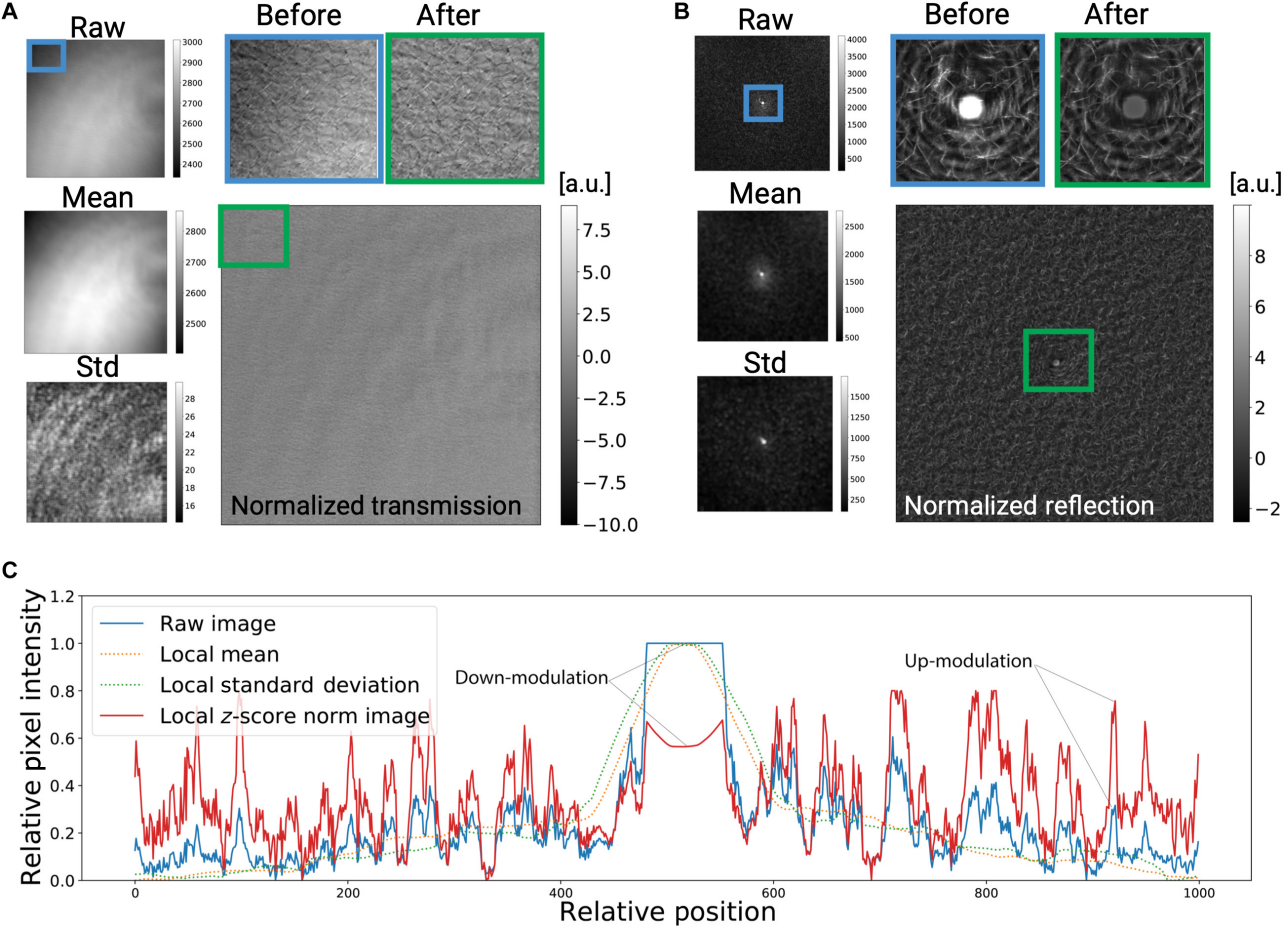
Intensity normalization. (A) Transmission image example: Left column shows raw image, mean, and standard deviation maps of an ROI; column 2 shows a normalized image (bottom) and two zoomed-in regions of interest for comparison between before (blue frame) and after (green frame) normalization. (B) RCM image example: Left column shows raw image, mean, and standard deviation maps, and top right shows a zoomed-in comparison between before (blue frame) and after (green frame) normalization, where we were able to tune down the intensity of the bright laser spot in the center of the field of view. (C) Intensity profiles comparing a reflection image before and after normalization. *Z* score shows the drop in saturation at the center of the image.

A normalization scheme is applied to all raw imaging data in order to equalize brightness in both the transverse and longitudinal planes as well as to enhance overall image contrast. Specifically, we use a sliding window approach to perform spatially localized *Z*-score normalization [34], as shown in Fig. 2. This technique transforms pixel intensities into dimensionless zero-mean distribution with a unit standard deviation, preserving spatial information while reducing light gradients across an image (e.g., Fig. 2A and B). In this study, we implement the sliding window kernel with a dimension of (1,101,101) and a stride of (1,1,1) such that the mean and standard deviation for any single window is calculated using a neighborhood of 10,200 pixels. Application of this kernel yields spatial arrays representing local means $\mu \left(x,y,z;c\right)$ and local standard deviations $\sigma \left(x,y,z;c\right)$. These local statistical distributions are used to perform *Z*-score transformation through pixel-wise subtraction and division on the raw images $R\left(x,y,z;c\right)$ according to Eq. 1, resulting in a normalized output $N\left(x,y,z;c\right)$ with locally and globally equalized pixel values (e.g., Fig. 2A and B). Of note, this method suppresses the bright central artifact common to RCM imaging (Fig. 2B) and prioritizes local variations over broader intensity gradients (Fig. 2C).

#### Patching and cohorts

After normalization, we separate the original data into image patches comprising smaller fields of view under the hypothesis that GT prediction only requires local contextual information. This approach maximizes available training data as each subvolume yields a separate sample for learning and also accommodates the practical limitations of GPU memory. To generate image patches, we divide the original matrix of size $\left(z,\;x=4096,\;y=4096,\;c\right)$ into $\left(z,\;x=256,\;y=256,\;c\right)$ patches, where *z* is the depth of the image and *c* is the number of input channels. By maintaining the original *z* dimension, we preserve *z*-oriented features, which in turn supports the reconstruction of fibers along the *z* axis. This protocol results in a total of 2,816 patches with dimensions of $\left(\text{batchsize},z=20,x=256,y=256,c\right)$ for training and 1,024 patches of identical dimensions for validation. For testing, we used an independent dataset with a size of $\left(z=150,x=512,y=512,c\right)$. To improve model generalizability, we incorporated data from 2 distinct fibrin scaffold samples for training and employed an independent third sample for hyperparameter tuning and validation. This approach increases the diversity of the training set, reducing within-sample correlations and enabling a more robust validation process. Finally, testing was performed using a fully independent fibrin scaffold sample distinct from both samples used during training and validation.

#### Network architecture

**Fig. 3.**
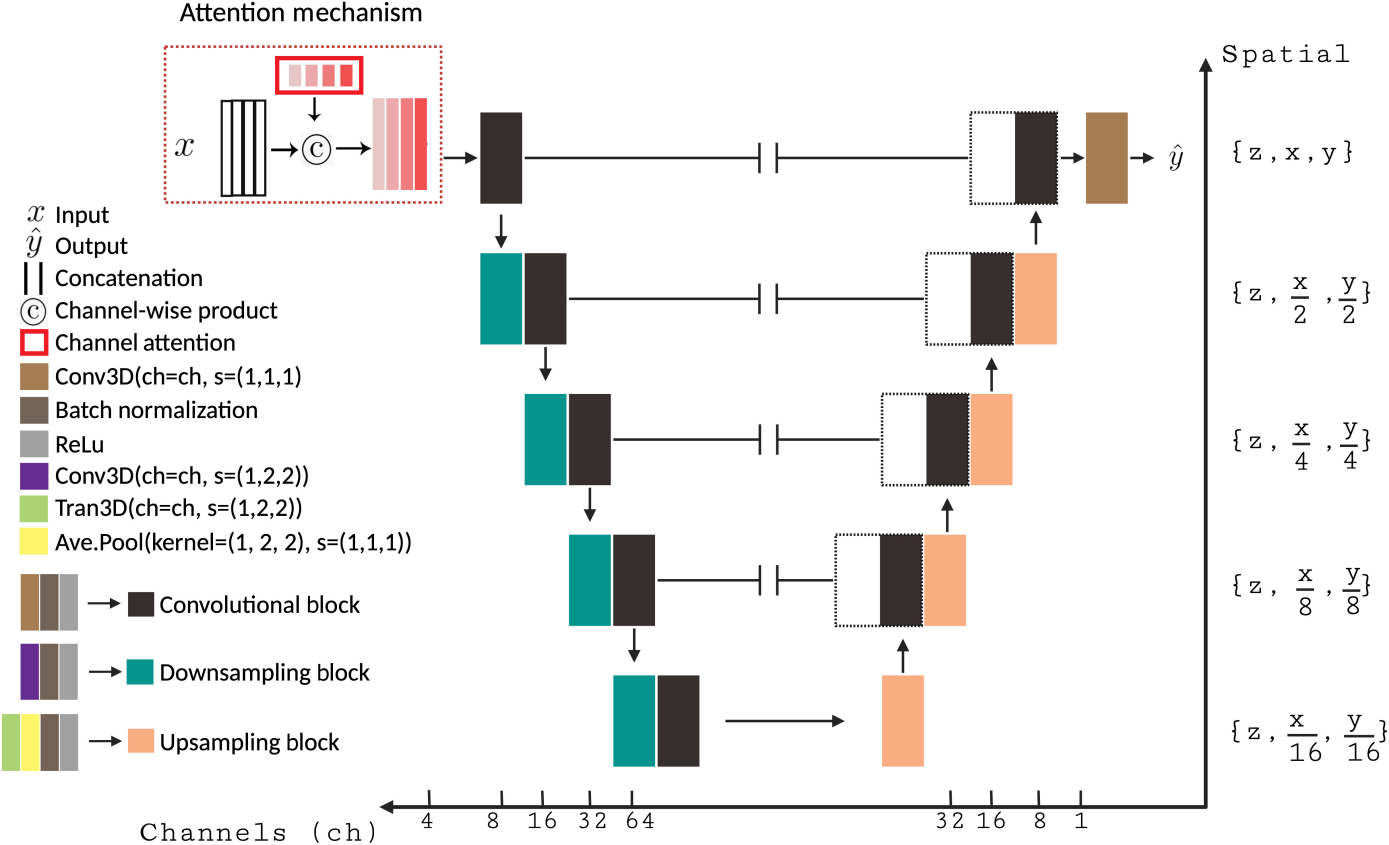
Network architecture: Overview of encoder–decoder (U-Net) network architecture for proposed image reconstruction task. For each resolution level, spatial dimensions are shown along the *y* axis (as a ratio of original data size), while channel depth is shown along the *x* axis. Note that within a block, information flows from left to right.

In this study, we implement an image-to-image mapping model using a fully convolutional encoder–decoder (U-Net) architecture. The encoder–decoder architecture is a standard approach for image-to-image mapping tasks and has been successfully adapted for similar previous work in microscopy [33,35–37]. Our fully convolutional encoder–decoder model is implemented using a series of convolutional blocks defined as the application of a 3D convolution operation, batch normalization, and rectified linear unit (ReLU) nonlinearity as shown in Fig. 3. A downsampling convolutional block is implemented by utilizing a (1,2,2) stride length in the convolution operation. Similarly, an upsampling convolutional block is implemented by replacing the standard convolution with a (1,2,2) stride length convolution transpose operation followed by a nonstrided average pooling operation with kernel size (1,2,2) to mitigate zero-filled upsampling artifacts. In total, our encoder is composed of a series of 4 alternating pairs of nonstrided and strided downsampling blocks followed by a symmetric decoder of 4 alternating pairs of nonstrided and strided upsampling blocks. With each downsampling operation, the feature map depth is doubled from an initial 4-channel input to a maximum feature map depth of 64. Refer to the Supplementary Materials for the mathematical definitions of the blocks.

We further introduce a per-channel attention mechanism at the first layer of the network, as shown in Fig. 3 (top left). This layer is designed not only to enhance network performance but also to enable the model to explicitly learn the relative importance of each input channel for optimal reconstruction, overall improving model interpretability. To implement this strategy, each channel is assigned a single trainable global weight scalar, $w_{{\mathrm{Ref}}_{0}},w_{{\mathrm{Ref}}_{1}},w_{{\mathrm{Ref}}_{2}},w_{\mathrm{Tra}}$, which is multiplied across all pixel values of each corresponding input channel data. During the optimization process, the model is allowed to increase or decrease the relative contribution of any individual channel to maximize reconstruction accuracy. Upon convergence, the learned per-channel weight values reflect the relative importance of each input to the final reconstruction task.

### Optimization

#### Loss function

Our loss function is composed of a combination of low-dimensional per-pixel error as well as high-dimensional structural consistency. The low-dimensional loss component $L\left(y,\hat{y}\right)$ is defined using a series of $L^{p}$ norm values, $L_{p}^{p}=\frac{1}{N}{\left\|.\right\|}_{p}^{p}$, with *p* = 1 and *p* = 3, as shown in Eq. 2, where *N* represents the total number of pixels within the image domain *I*, while i,j,k are indices corresponding to the spatial dimensions x,y,z. Generally, higher values of *p* more effectively penalize outlier errors, which can accelerate convergence. However as the model converges, the partial derivative of higher-order loss terms asymptotically approaches zero, which in turn degrades gradients used for optimization. Furthermore, high-order norm functions prioritize a uniform distribution of error at the expense of precise predictions, resulting in blurring at the edges of reconstructed images. The *p* = 1 term, on the other hand, encourages both improved gradient stability during optimization as well as a sparse distribution of errors, overall preserving finer details during image-to-image regression tasks. The high-dimensional structural consistency loss component is defined using structural similarity $\text{SSIM}\left(y,\hat{y}\right)$, a term that captures more abstract perceptual differences within a local neighborhood of pixels [38]. Together, the $L_{1}$, $L_{3}$, and *SSIM* terms are combined using weights α and β to adjust the contribution of $L_{3}$ and *SSIM* relative to the main $L_{1}$ component. After a grid search, we determined that $\alpha =10^{-1}$ and $\beta =10^{-4}$ yielded optimal performance (Eq. 2). Optimization is performed using the Adam method [38] at a learning rate of $\mathrm{LR}=10^{-4}$ and a batch size of 5.

#### Implementation details

All experiments were written using TensorFlow 2.5.0 within the Python 3.8.5 environment. Model training was performed on systems featuring 4 NVIDIA GeForce RTX 2080 Ti GPUs (11 GB of VRAM), a 16-core AMD Epyc processor 3.0 GHz, 256 GB of DDR4 RAM, and a 960 GB NVMe SSD.

### Impact of image modality on model performance

#### Per-channel analysis

To investigate the contribution of different input channels to model performance and determine how channels complement one another, we conduct an input channel ablation study. In addition to the baseline experiment with all input channels, we perform experiments isolating the input to just transmission or reflection groups. This procedure yields a total of 3 different input permutations:

• *Tra* + *Ref*: Training the model with both transmission and RCM.

• *Tra*: Training with only transmission.

• *Ref*: Training with only RCM.

In addition to statistical metrics of reconstruction accuracy, we generate postprocessed image error maps to highlight salient features in the prediction ($\hat{y}$) relative to GT (*y*). To generate these maps, we first threshold the original images and perform a *z* projection to create a binary mask of relevant fiber structures. This step is performed using Fiji software (https://imagej.net/software/fiji/). Then, we multiply raw images ($\hat{y}$ and *y*) by the binary fiber mask followed by a signed error difference calculation that we refer to as $\hat{y}-y$ for simplicity. In the resulting error map, any positive value indicates model overestimation, while any negative value indicates model underestimation.

#### Transmission channel blurring

Transmission channel resolution is degraded with thicker and higher concentration of ECMs because of light scattering. To assess the effect of this potential source of quality degradation, such ECMs are simulated computationally by applying a 5 × 5 Gaussian blur kernel of different standard deviations: 1, 3, or 5. These blurred transmission images were combined with all 3 raw (unchanged) RCM inputs, yielding an additional 3 ablation experiments:

• *Tra*, σ=1: Transmission blurred by a 5 × 5 kernel with σ = 1.

• *Tra*, σ=3: Transmission blurred by a 5 × 5 kernel with σ = 3.

• *Tra*, σ=5: Transmission blurred by a 5 × 5 kernel with σ = 5.

#### RCM wavelength comparison

To evaluate the relative contributions of each RCM image acquired at different excitation wavelengths, we trained models using distinct wavelength-specific image sets in conjunction with the transmission image. The goal of these experiments is to determine the extent to which each wavelength contributes to predictive accuracy of fibrillar parameters, such as fiber count, length, and width. The following experiments were trained and assessed to give us insight on whether all 3 RCMs are necessary for robust fibrillar characterization: 

• *Tra + Ref*405: Training with transmission and the 405-nm RCM.

• *Tra + Ref*488: Training with transmission and the 488-nm RCM.

• *Tra + Ref*561: Training with transmission and the 561-nm RCM.

### Robustness of best-performing model

One possible issue that can affect model performance is the presence of interference rings inherent to the RCM modality, especially near the glass interface. Acknowledging this potential source of error, we deliberately ensured that the training cohort comprised images at the glass interface containing this particular artifact pattern, allowing the model to learn high-quality mappings even in the presence of image degradation. To test for robustness to this known artifact, we carefully interrogated model predictions specifically in regions exhibiting these ring patterns, with a particular emphasis on evaluating the consistency of fiber predictions in the central region where the rings appear.

Furthermore, we assess out-of-distribution model generalizability on higher-density samples (5 and 10 mg/ml) where pore size and fibrillar structure significantly differ from those in the lower-density training dataset (2.5 mg/ml). For this evaluation, we generate predictions from our top-performing model without additional fine-tuning on 2 new 3D image stacks of 5 and 10 mg/ml fibrinogen concentration of dimension 40 × 40 × 6 μm^3^ with a pixel size of 77.69 nm, a pixel dwell time of 2 μs, and a step size of 100 nm.

### Statistical analysis

#### Nonstructural analysis

Model reconstruction error is estimated using mean squared error (MSE; Eq. 3), structural similarity index (SSIM; Eq. 4), peak signal-to-noise ratio (PSNR; Eq. 5), and Spearman’s rank correlation coefficient (ρ; Eq. 6). Definitions of these metrics and Eqs. 3 to 6 are in the Supplementary Materials. Prior to metric derivation, all images and model predictions are normalized to a distribution between [0,1]. Metric variance is estimated using a bootstrapping technique implemented using 10,000 overlapping patches from the test cohort with dimensions of (batchnumber, *z* = 15, *x* =100, *y* =100, c).

#### Structural analysis

**Fig. 4.**
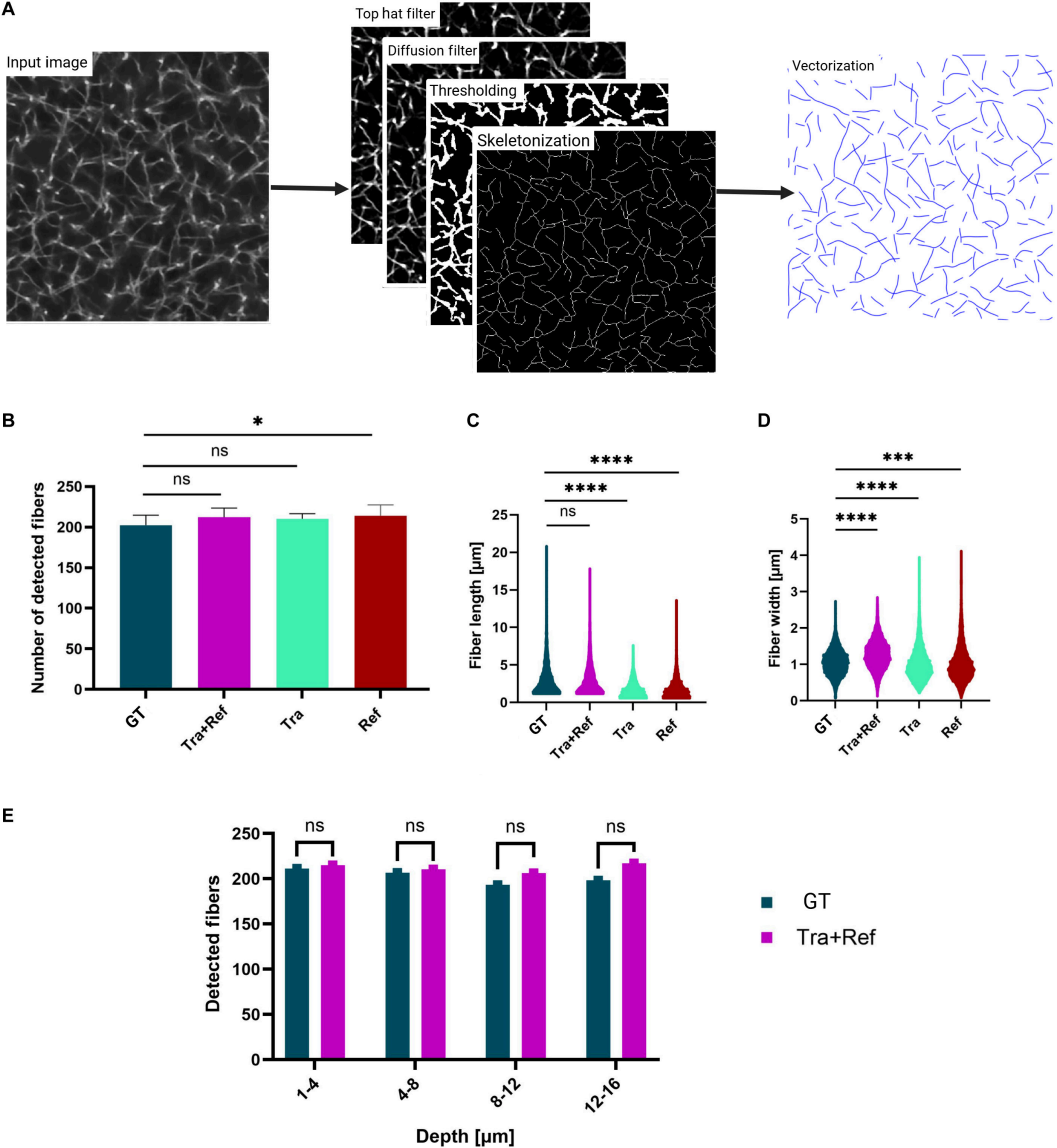
Structural analysis. (A) Example of the process used to analyze fibers using GTFiber. A series of filters are applied to the raw image, followed by a vectorization step [39]. Distributions per slice of number of detected fibers (B), fiber length (C), and fiber width (D) for GT, *Tra + Ref*, *Tra*, and *Ref* predictions. (E) Number of detected fibers within an image volume, binned by depth.

The ability of model reconstructions to retain relevant semantic meaning compared to GT was assessed using GTFiber, an open-source software package with algorithms to track and analyze polymers, filaments, and fibrous samples [39]. In this study, GTFiber is used to produce a vectorization map of the fibers (Fig. 4A) to calculate total number of fibers (Fig. 4B), fiber length (Fig. 4C), and width (Fig. 4D) within a volume of dimension 40 × 40 × 16 μm^3^. Specific application of GTFiber to a dataset requires manual configuration of various filters to the input image according to the specified parameters shown in Fig. S2. For this analysis, identical parameters were used for both GT and prediction. Prior to application of GTFiber software, GT images were denoised using the spatial redundancy subsampling method proposed by Li et al. [40]. Refer to Fig. S3 for an example. All statistical analyses were carried out in GraphPad Prism version 10.2.3 for Windows (GraphPad Software, Boston, MA, USA; www.graphpad.com). A 2-tailed paired parametric *t* test was used with a level of significance, α=0.05.

## Results

### Quantitative analysis

**Fig. 5.**
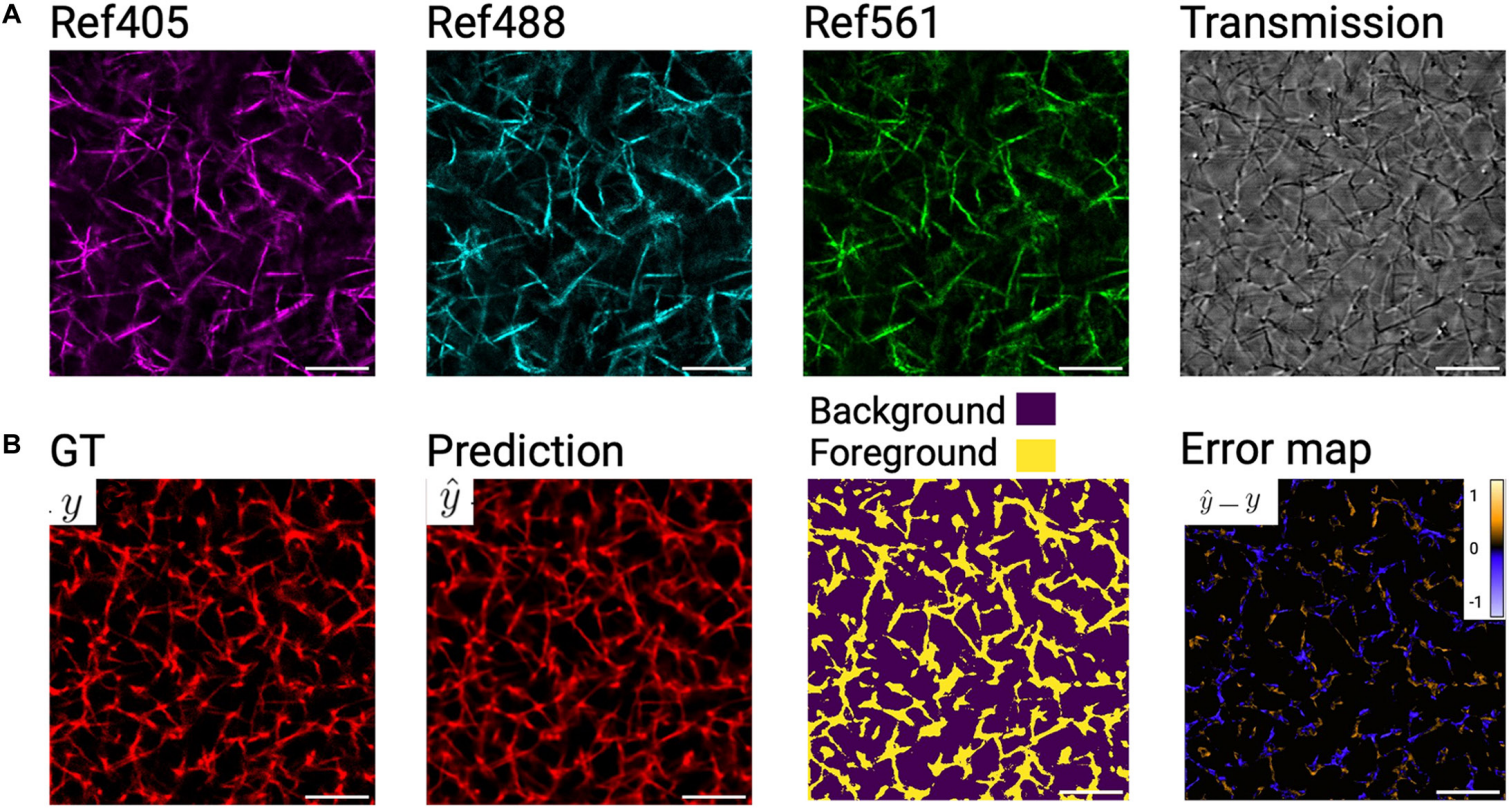
Example *Tra + Ref* prediction. (A) *Tra + Ref* inputs of a selected ROI comprising RCM at 3 wavelengths (405/488/561 nm) and a laser scanning transmission image. (B) Corresponding fluorescently labeled image (GT = *y*), model prediction ($\hat{y}$), binary fiber mask, and a normalized error map ($\hat{y}-y$) created according to Per-channel analysis. Note that scale bars are drawn at 10 μm.

A summary of results across all input channel permutations is shown in Table 1, with reconstruction accuracy characterized by normalized MSE, SSIM, PSNR, and Spearman coefficient. We notice that *Tra + Ref* performed the best across all 4 metrics with $\mathrm{MSE}=0.3542*10^{-2}$, SSIM=0.7891, pSNR=24.5469, and Spearman correlation coefficient ρ=0.8782. Figure 5 shows a randomly selected slice of *Tra + Ref* output demonstrating the quality of GT prediction. Figure 5A shows the original input channels for model prediction, while Fig. 5B shows corresponding GT and generated reconstructed outputs; notice that numerous fine details including fiber structure are recovered. Review of the generated error map suggests that discrepancies in model predictions are primarily distributed along the edges of existing fibers, resulting in over- or underestimation of fiber intensity and width. Importantly, we did not observe significant hallucinations or other de novo generation of completely new fiber structures in *Tra + Ref* reconstructions. A similar analysis was completed for models *Tra* and *Ref* (Fig. S4) showing hallucinations and missing nodes.

**Table 1.** Quantitative analysis of the trained models *Tra* + *Ref*, *Tra*, and *Ref*. This table presents the mean and standard deviation for MSE, SSIM, PSNR, and Spearman’s rank correlation of the 3 different models explained in Per-channel analysis.

Model	MSE (*10^−2^)	SSIM	PSNR (dB)	Spearman
*Tra* + *Ref*	0.3542 ± 0.0487	0.7891 ± 0.0229	24.5469 ± 0.5862	0.8782 ± 0.0265
*Tra*	0.4020 ± 0.0478	0.7614 ± 0.0265	23.9886 ± 0.5144	0.8577 ± 0.0308
*Ref*	0.7306 ± 0.1151	0.6954 ± 0.0337	21.4172 ± 0.6868	0.6376 ± 0.0767

As motivated in Per-channel analysis, a per-channel attention mechanism is used to learn the relative contributions of each input channel for optimal reconstruction accuracy. Upon model convergence, the weight associated with the transmission channel, $w_{\mathrm{Tra}}$, surpassed the weights associated with reflection channels, $w_{{\mathrm{Ref}}_{0}},w_{{\mathrm{Ref}}_{1}},w_{{\mathrm{Ref}}_{2}}$, by nearly 40%, highlighting the relative importance of the transmission channel in the reconstruction task. As noted in Table 1, ablation experiments suggest a similar trend with *Tra* outperforming *Ref* for all reconstruction error metrics. A comparison of model predictions for *Tra* and *Ref* as shown in Fig. S4 further affirms this observation with significant degradation in performance when transmission data are withheld from the model.

The effect of a blurred transmission image on prediction performance was assessed by models *Tra*, σ=1, *Tra*, σ=3, and *Tra*, σ=5 (results shown in Table 2). In these ablation experiments, we observed progressive decline in performance with increasing σ. Corresponding images are shown in Fig. S5. Of note, the loss of information with blurred transmission images does not appear to be mitigated by RCM images, which remained intact for these experiments; indeed, these results further confirm the critical importance of transmission images for robust reconstruction.

**Table 2.** Quantitative analysis of the trained models *Tra* + *Ref*, *Tra*, σ = 1, *Tra*, σ = 3, and *Tra*, σ = 5. This table presents the mean and standard deviation for MSE, SSIM, PSNR, and Spearman’s rank correlation of the 4 different models explained in Transmission channel blurring.

Model	MSE (*10^−2^)	SSIM	PSNR (dB)	Spearman
*Tra* + *Ref*	0.3542 ± 0.0487	0.7891 ± 0.0229	24.5469 ± 0.5862	0.8782 ± 0.0265
*Tra*, σ = 1	0.3882 ± 0.0573	0.7767± 0.0253	24.1556 ± 0.6359	0.8642 ± 0.0292
*Tra*, σ = 3	0.4770 ± 0.0627	0.7543 ± 0.0269	23.2515 ± 0.5676	0.8516 ± 0.0287
*Tra*, σ = 5	0.5660 ± 0.0747	0.7209 ± 0.0310	22.5092 ± 0.5703	0.8047 ± 0.0397

We further explored the contribution of each RCM wavelength to model accuracy. Results of the models mentioned in RCM wavelength comparison are presented in Table 3. Among the individual wavelength models, *Tra + Ref*405 shows performance closest to the full model (*Tra + Ref*). In contrast, *Tra* + *Ref*488 and *Tra + Ref*561 exhibit a progressive decline in metrics, with the 561-nm model performing the worst.

**Table 3.** Quantitative analysis of the trained models *Tra* + *Ref*, *Tra* + *Ref*405, *Tra* + *Ref*488, and *Tra* + *Ref*561. This table presents the mean and standard deviation for MSE, SSIM, PSNR, and Spearman’s rank correlation of the 3 different models explained in RCM wavelength comparison.

Model	MSE (*10^−2^)	SSIM	PSNR (dB)	Spearman
*Tra* + *Ref*	0.3542 ± 0.0487	0.7891 ± 0.0229	24.547 ± 0.586	0.8782 ± 0.0265
*Tra* + *Ref*405	0.3505 ± 0.0484	0.7826 ± 0.0236	24.594 ± 0.589	0.8701 ± 0.0283
*Tra* + *Ref*488	0.3778 ± 0.0491	0.7683 ± 0.0269	24.264 ± 0.566	0.8563 ± 0.0303
*Tra* + *Ref*561	0.4012 ± 0.0608	0.7663 ± 0.0250	24.014 ± 0.640	0.8649 ± 0.0286

### Structural analysis

We utilized GTFiber to assess the fibrillar similarities and differences between GT and the predictions of our best-performing model, *Tra + Ref*. Results are presented in Fig. 4. Overall, *Tra + Ref* shows no significant difference as compared to GT in number of fibers detected per slice (Fig. 4B) or fiber length (Fig. 4C). However, a small but statistically significant difference is observed in fiber width (0.21 ± 0.01 μm), with model reconstructions showing slightly thicker fibers (Fig. 4D). Given the systematic nature of this error (likely due to the $L_{3}$ loss function component as detailed in Loss function), we note that application of a sharpening filter successfully eliminates this difference in width (Fig. S5C and E). We further analyzed the number of detected fibers across a depth of 16 μm by grouping the data into depth intervals of 1 to 4, 4 to 8, 8 to 12, and 12 to 16 μm. A 2-tailed paired *t* test shows no significant difference between GT and the prediction of *Tra + Ref* (Fig. 4E).

Compared to our baseline *Tra + Ref*, *Tra* showed no significant difference in the number of fibers detected per slice; however, significantly shorter and thicker fibers were predicted with 2.31 ± 2.32 and 0.51 ± 0.55 μm (median ± SD), respectively (Fig. 4B to D). By contrast, *Ref* showed significant differences in the number of detected fibers per slice (11.63 ± 6.23 fibers), fiber length (1.39 ± 0.28 μm), and fiber width (0.41 ± 0.59 μm) compared to the GT (Fig. 4B to D). The underperformance of *Ref* can be attributed to the nature of RCM images, which often fail to capture fibers oriented along the *z* axis due to insufficient light reflection. These results demonstrate that transmission images, either alone or in combination with RCM images, can reliably reproduce GT fibrillar structure as observed in Fig. 4C. These findings corroborate our results in Table 1, where combining transmission images with RCM to train the model produced the best results.

We further examined the impact of individual RCM wavelengths on fiber detection, length, and width predictions. As shown in Fig. S6, all models incorporating RCM images maintained a comparable number of detected fibers per slice with no significant differences observed, except for *Tra + Ref*488 (Fig. S6A). However, fiber length predictions exhibited significant deviations from GT across all single RCM-based models (Fig. S6B). This effect is particularly pronounced in the 488- and 561-nm models, which show the greatest discrepancies. Similarly, fiber width predictions were significantly affected by RCM wavelength selection, with all models predicting systematically different fibers than GT (Fig. S6C). The widening effect is most pronounced in the 561-nm model, suggesting that longer wavelengths may introduce artifacts that impact fiber morphology estimation. These findings support our quantitative analysis (Table 3), highlighting the importance of including all 3 RCM images as an input to preserve fibrillar structure.

### Qualitative comparison

**Fig. 6.**
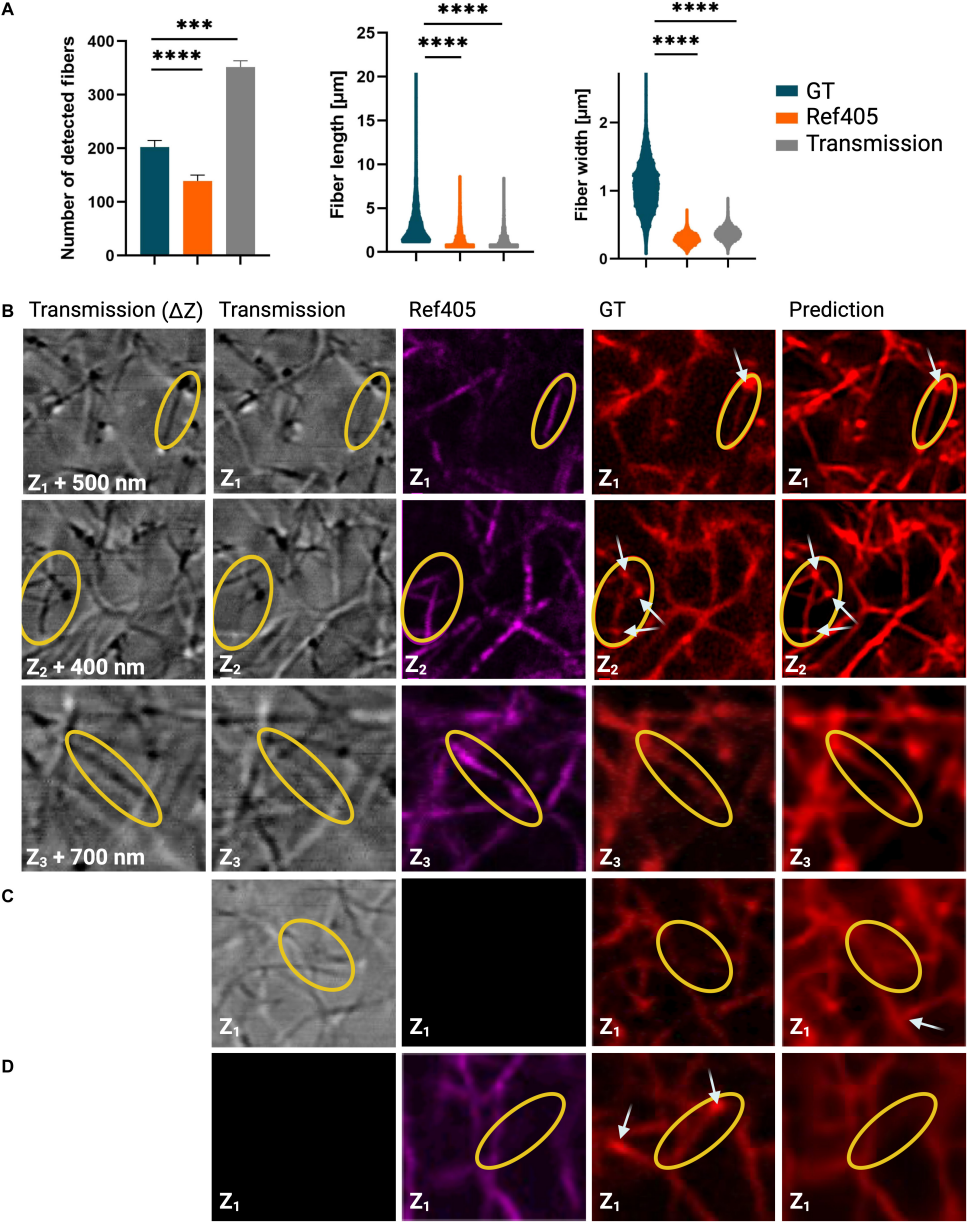
Limitation and cooperation between input image modalities. (A) GTFiber analysis of number of fibers per slice, fiber length, and width within a fibrin volume using the 3 imaging modalities: fluorescence (GT), RCM example (405 nm), and transmission. (B) Three *Tra + Ref* examples (one per row) showing a first transmission slice at depth $Z_{n+\text{offset}}$ and second at $Z_{n}$, as well as RCM (405 nm), GT, and prediction at $Z_{n}$. Yellow ovals highlight features inconsistent between image types. White arrows point to nodes. Corresponding image types for *Tra* (C) and *Ref* (D).

To better characterize the complimentary information present across transmission and RCM input images, each individual imaging modality is analyzed using GTFiber within a volume of dimension 40 × 40 × 16 μm^3^. One key observation is the notable increase in number of detected fibers in the transmission channel as compared to GT, which is expected because transmission is not a confocal modality and thus it images a thicker focal volume (Fig. 6A). Also, as discussed above, RCM is insensitive to out-of-plane fibers and thus has a lower fiber count. Further, both fiber length and width in either of these modalities deviate largely from GT and are insufficient for network reconstruction (Fig. 6A).

The ability of *Tra + Ref*, *Tra*, and *Ref* to predict all fibers and nodes in an image is exemplified in Fig. 6B to D. Interestingly, while the transmission channel was heavily weighted during training, we did observe a *Z*-coordinate offset for each fiber, with the offset step size changing across the field of view without any detectable trend. Columns 1 and 2 in Fig. 6B show examples of this offset that ranges from 400 to 700 nm. When examining the reflection, GT, and prediction images, we notice that features in the GT image align with those in $Z_{n+\text{offset}}$, not the corresponding $Z_{n}$ transmission image. Unexpectedly, the *Tra + Ref* predicted image contains features that are not in focus within the corresponding transmission image (column 2), but are in focus in the parfocal reflection image (column 3). *Tra* prediction hallucinated fiber structure (Fig. 6C, yellow ovals), while *Ref* failed to detect fibers (Fig. 6D, yellow ovals). Furthermore, *Tra + Ref* retrieved all nodes (Fig. 6B), whereas *Tra* hallucinated nodes (Fig. 6C, white arrow); conversely, *Ref* failed to detect several nodes that were present in GT (Fig. 6D, white arrows). To test if the transmission image focal plane offset was an artifact of the microscope condenser alignment position, we shifted the condenser along the optical axis but found no change in the location of features. This result suggests that the observed focal plane offset is inherent to the laser scanning transmission modality.

## Discussion

Here, we present a technique to image ECM fibers that frees up a fluorescent channel typically dedicated to that task, where the said channel is better used for molecular labeling. Our approach exclusively uses the label-free modalities of transmission and RCM while compensating for their limitations as previously supported by Fig. 6A. Specifically, *Tra + Ref* compensates for the RCM limitation of not detecting a significant portion of fibers and the poor optical sectioning inherent to transmission microscopy. Further, *Tra + Ref* successfully reconstructs scaffold structure, which is valuable for real-time deformation analysis in the study of cell–ECM interactions. Structural and statistical comparisons between the GT and predictions show strong agreement in number of detected fibers and length. However, the predicted fibers were slightly wider than their GT counterparts, likely due to a spectral bias in CNNs that prioritizes low-frequency features during the optimization process [41]. Such a bias makes it challenging for the network to reconstruct high-frequency features such as the sharp edges of fibers with high precision. Additionally, $L_{p}$ norm-based losses, which are known to induce blurriness in structures [42], contribute to a disproportionate increase in width relative to length, as fibrin fibers are elongated structures. Importantly, however, the shape of fiber width distribution is similar between the predicted and GT data in our best performing model (Fig. 4D), overall suggesting a systematic bias that can be compensated for using postprocessing techniques such as a sharpening filter (Fig. S5) or morphologic erosion. As needed, a calibration step could be introduced to determine optimal postprocessing hyperparameters that best remove this artifact based on dataset-specific properties.

We further assessed the reconstruction error of our models using metrics including MSE, SSIM, PSNR, and Spearman correlation, as summarized in Table 1. Each of these metrics captures image similarity from a different perspective, and they should be interpreted holistically within the context of the specific task rather than in isolation. MSE reflects a low-dimensional per-pixel accuracy without regard for spatial distribution of error. In a normalized image with pixel intensities ranging from 0 to 1, MSE values on the order of ~10^−2^ as exhibited by our models suggest that the average quadratic deviation per pixel is approximately 1% of the maximum image intensity, which we consider a reasonable outcome for this task. SSIM is a normalized metric with a range of [0,1], with higher SSIM values indicating statistical similarity accounting for spatial distribution on a patch-by-patch basis. Our top model yielded SSIM values of ~0.79, which is comparable to other image-to-image mapping tasks. In a previous study [33], a model for virtual staining of hematoxylin and eosin (H&E) images achieved an SSIM of ~0.725.

The Spearman correlation coefficient (ρ) is a normalized metric with a range of [0,1], with higher values indicating a direct, monotonic global relationship between image pairs without consideration of absolute pixel values. We found ρ between *Tra + Ref* prediction and GT to be ~0.87, overall suggesting strong correlation. Finally, PSNR is a metric that characterizes image noise on a logarithmic scale measured in decibels. In image reconstruction tasks, typical PSNR values fall within the 20- to 25-dB range, with higher values representing better image quality. Our model predictions yield PSNR values of approximately 24.5 dB and are overall similar to PSNR values of the GT. Overall, the collective interpretation of these metrics, alongside previously established benchmarks in related work ([32,33,36]), supports the conclusion that our model is capable of generating accurate and robust reconstructions.

While training with the single transmission and 3 RCM channels produced a well-performing model, we did question whether both reflection and transmission channels were necessary. Our ablation experiments (Per-channel analysis) provide insight into the contribution of each input channel in our best-performing model (*Tra + Ref*). Interestingly, while the transmission channel had greatest weight during training, a second model trained on the transmission alone (*Tra*) predicts shorter and narrower fibers as compared to GT and thus does a poor job reconstructing the GT mesh structure. A third model trained on the RCM channels alone (*Ref*) had similar limitations.

Lastly, we explored robustness of *Tra + Ref* to ECMs having increased thickness and turbidity, 2 factors that are dependent upon the design of a tissue culture experiment. As expected, dynamically blurred training sets diminished prediction accuracy (Table 2 and Fig. S5C), but application of a sharpening mask (radius of 4 pixels, mask weight of 0.6) can reasonably recover fiber contrast and resolution (Fig. S5, C as compared to E). This suggests that our tool could be beneficial for thicker samples than we imaged here, but a further developed model may become necessary beyond some thickness threshold.

As expected, RCM could not detect all fibers in the focal plane due to dependency on fiber axial orientation. However, one surprising observation is the focal plane mismatch between transmission and GT images as assessed by the appearance of fibers. In fact, we found that the difference in focal plane was on the order of 0.5 μm and was not uniform between regions of interest. This depth of mismatch is significant for reconstructing and understanding ECM mesh architecture in the context of tissue engineering, and thus needs to be accounted for. Fortuitously, while the abundance of spatial information in the transmission channel greatly aided the model in predicting fibers, it appears that the RCM channels can “pull” fibers back into the GT focal plane, thus compensating for the transmission offset. Further, because the focal offset seems to be dependent on fiber orientation, local fiber density, and sample depth, a simple offset applied to all region of interest (ROI) is insufficient for accurate prediction. This claim is supported by the fact that *Tra* exhibits hallucinated fibers and nodes, and required the RCM to both define the true focus and provide optical sectioning (Fig. 6C).

We further assess model generalizability and robustness to image artifact as well as out-of-distribution conditions (Fig. S7). First, we analyzed predictions on 2.5 mg/ml samples in regions where RCM images contained diffraction rings due to proximity to the glass interface. Despite the presence of these artifacts, *Tra + Ref* was able to accurately reconstruct scaffold fibers, demonstrating resilience to diffraction-induced distortions (Fig. S7A). Indeed, a key advantage of neural networks is the capacity to recover high-quality mappings from any representative distribution of input and output image pairs, including input images degraded by various artifacts. In other words, a model can implicitly learn to reconstruct artifact-free images directly from artifact-degraded inputs without the need to manually compensate for data corruption so long as the sources of image degradation are adequately represented in the training dataset and do not irreversibly damage the underlying signal of interest. Second, we observed that the top-performing model was able to recover fibers even in samples of higher density (5 and 10 mg/ml) than those used in training (2.5 mg/ml), although overall performance in this out-of-distribution context is lower than in the original baseline experiments. We suspect that this is at least in part due to degradation of overall signal across the input images, an observation most evident in the 10 mg/ml condition where fiber contrast and subtle finer structure appears significantly diminished across both input and ground-truth channels (Fig. S7B). This suggests that the underlying theoretical upper bounds of fiber reconstruction may be lower for higher density samples regardless of technical approach. Nonetheless, we acknowledge that further optimization in this setting may be best achieved using a dedicated model to accommodate the broader range of pore sizes and fiber arrangements.

We plan to extend model development to include predictions of molecules within and surrounding cells where GT includes, for example, fluorescent labels, such as 4′,6-diamidino-2-phenylindole (DAPI), phalloidin, and lectin, which stain cell nuclei, actin fibers, and endothelial cells, respectively. This will enable longitudinal studies without the need for invasive fixation and staining procedures, preserving the integrity of experiments. We hypothesize that complementary information exists in the images captured by different microscopy modalities, and any well-trained model should be capable of extracting this information to virtually label specific structures of interest. In addition, we plan to explore alternative architectures such as generative adversarial networks (GANs) and incorporate perceptual loss functions. These methods have demonstrated success in similar tasks [33,36,37] and in superresolution applications [43,44], effectively performing image-to-image mapping tasks and enhancing sharp borders with the goal of precisely replicating fiber width.

### Conclusion

In this paper, we present a method for virtually staining fibrin fibers using a 3D CNN, with label-free RCM and transmission images as inputs and fluorescently labeled images as outputs. Our best-performing model, *Tra + Ref*, successfully reconstructs the scaffold structure, recovers missing fibers from reflection data, and enhances optical sectioning. We began by acquiring the training dataset using multiple microscope modalities and validated the model with a blind test, assessing predictions through quantitative, qualitative, and structural analyses. Our results demonstrate that both RCM and transmission data are essential for optimal model performance, where RCM provides parfocality. The complementary nature of these modalities arises from the volumetric information captured in the image stacks, underscoring the importance of 3D data for accurate predictions. However, some blurriness appeared in the synthetic images, which we attribute to limitations in the CNN-based reconstruction and the associated loss functions. These limitations led to small discrepancies in fiber width between the fluorescent GT and the predicted images, which were correctable to an extent via postprocessing filters. Overall, the prediction and GT images show strong agreement in fiber detection and length. We have made this tool open access, enabling its integration with any commercially available laser scanning confocal microscope.

$$\begin{array}{c} N\left(x,\;y,\;z;\;c\right)=\frac{R\left(x,\;y,\;z;\;c\right)-\mu \left(x,\;y,\;z;\;c\right)}{\sigma \left(x,\;y,\;z;\;c\right)} \end{array}$$ (1)

$$\begin{array}{c} L\left(y,\hat{y}\right)=\frac{1}{N}\sum_{\mathrm{ijk}\in I}\left(\left|y_{ijk}-{\hat{y}}_{ijk}\right|+\alpha {\left|y_{ijk}-{\hat{y}}_{ijk}\right|}^{3}\right)\\ +\beta \left(1-\text{SSIM}\left(y,\hat{y}\right)\right). \end{array}$$ (2)

## Data Availability

The source code and data are available at: https://github.com/AndresFGuerrero/Label-free-prediction-of-fluorescently-labeled-fibrin-networks/

## Supplementary Materials

Supplementary 1 Supplementary Text Figs. S1 to S7 References [46,47]
