# Supplementary material for: Label-Free Prediction of Fluorescently Labeled Fibrin Networks
Source: Biomater Res. 2025 May 28;29:0211. doi: 10.34133/bmr.0211 (PMC12117218; doi:10.34133/bmr.0211)
Supplement: Supplementary 1 — Supplementary Text Figs. S1 to S7 References [46,47] [file bmr.0211.f1.pdf]

# Label-free prediction of fluorescently labeled fibrin networks Supplementary Materials

Sarah Eldeen[1*†], Andres Felipe Guerrero Ramirez[1,2†], Bora Keresteci[3], Peter Chang[2,*], and Elliot Botvinick[3,4,5,6,*]

[1]Department of Mathematical, Computational, and Systems Biology, University of California, Irvine, USA.

[2]Department of Radiological Sciences and Computer Sciences, University of California, Irvine, USA.

[3]Department of Biomedical Engineering, University of California, Irvine, USA.

[4]Beckman Laser Institute and Medical Clinic, University of California at Irvine, Irvine, USA

[5]Edwards Lifesciences Foundation Cardiovascular Innovation and Research Center, University of California, Irvine, Irvine, USA

[6]Department of Surgery, University of California, Irvine, Irvine, USA

[*]Address correspondence to: ebotvini@uci.edu, changp6@hs.uci.edu

[†]These authors contributed equally to this work.

## Statistical Metrics

- Mean Square Error (MSE): We normalize $y$ and $\hat{y}$ to be within a 0 to 1 domain, and implement the MSE calculation as it is used in the `skimage.metrics` sublibrary. With $i$ representing the $i^{th}$ pixel of any given image and $N$ representing the total number of pixels, equation is as follows:

$$MSE(y, \hat{y}) = \frac{1}{N} \sum_{i=0}^{N} (y_i - \hat{y}_i)^2 \tag{3}$$

- Structural similarity index measure (SSIM): Given a ground truth $S_y$ image and its corresponding predicted version $S_x$, we implement the SSIM calculation as built-in in the TensorFlow library, which in turn is based on the definition of Zhou Wang et. al. [47]:

$$SSIM(S_x, S_y) = \frac{(2\mu_x\mu_y + C_1)(2\sigma_{xy} + C_2)}{(\mu_x^2 + \mu_y^2 + C_1)(\sigma_x^2 + \sigma_y^2 + C_2)} \tag{4}$$

  where $\{\mu_x, \mu_y\}$ and $\{\sigma_x, \sigma_y\}$ are the means and variances of $S_x$ and $S_y$, respectively. $\sigma_{xy}$ is the covariance of $S_x$ and $S_y$. The two constants $C_1$ and $C_2$ are defined as $C_1 = (k_1L)^2$ and $C_2 = (k_2L)^2$. We use the built-in default values for $k_1 = 0.01$, and $k_2 = 0.03$, and $L = 1$.

- Peak Signal-to-Noise Ratio (PSNR): The Peak signal-to-noise ratio (PSNR) is the ratio between the maximum possible power of an image $S_y$ and the power of corrupting noise that affects the

quality of its representation $S_x$. The PSNR is defined as the ratio of the square of the largest possible pixel value $L = 1$ and the root mean square error $MSE(S_x, S_y)$

$$PSNR = 10 \log_{10} \left( \frac{L^2}{MSE(S_x, S_y)} \right) \tag{5}$$

- Spearman Correlation: The Spearman correlation assesses how well the relationship between two variables can be described using a monotonic function. We used the `scipy` sublibrary `stats` built-in function `stats.spearmanr`. The Spearman correlation coefficient $\rho$ can be defined as:

$$\rho = 1 - 6 \frac{\sum_{i=1}^{N} \left( R(S_{x_i}) - R(S_{y_i}) \right)^2}{N(N^2 - 1)} \tag{6}$$

Where $R(S_{x_i})$ denotes the rank of the $i^{th}$ element in $S_x$, and $N$ is the total number of pixels.

## Mathematical Definitions

- ReLU (Rectified Linear Unit): The ReLU function is defined as:

$$ReLU(x) = \max(0, x) \tag{7}$$

with $x$ being the input to the ReLU function. The function outputs $x$ if $x$ is positive, and 0 if $x$ is negative or zero.

- Batch Normalization Layer (BatchNorm): The Batch Normalization operation normalizes the output of a layer by adjusting and scaling the activations. For a mini-batch of activations $\mathbf{x} = \{x_1, x_2, \ldots, x_m\}$, the batch normalization is computed as follows [48]:

1. Calculate the mean and variance

$$\mu_{\text{batch}} = \frac{1}{m} \sum_{i=1}^{m} x_i \tag{8}$$

$$\sigma^2_{\text{batch}} = \frac{1}{m} \sum_{i=1}^{m} (x_i - \mu_{\text{batch}})^2 \tag{9}$$

2. Normalize the batch through Z-score

$$\hat{x}_i = \frac{x_i - \mu_{\text{batch}}}{\sqrt{\sigma^2_{\text{batch}} + \epsilon}} \tag{10}$$

where $\epsilon$ is a small constant added for numerical stability.

3. Scale and shift

$$y_i = \gamma \hat{x}_i + \beta \tag{11}$$

where $\gamma$ is the scale parameter, $\beta$ is the shift parameter, and $y_i$ is the final output after batch normalization.

- Conv3D (3D Convolution): Conv3D is a convolution operation that works on 3D spatial data. The

mathematical definition of a 3D convolution is given by:

$$y[i,j,k] = \sum_{d=0}^{D-1} \sum_{h=0}^{H-1} \sum_{w=0}^{W-1} x[i+d, j+h, k+w] \cdot w[d,h,w] + b \tag{12}$$

where: $x$ is the input 3D volume of size $(I_x, I_y, I_z)$, $w$ are the learnable parameters of the 3D kernel (filter) of size $(D, H, W)$., $b$ is the bias term, $y[i,j,k]$ is the output at position $(i,j,k)$, and $D$, $H$, and $W$ describe the size in terms of depth, height, and width of the kernel, respectively.

- Average Pooling: Average Pooling is a down-sampling operation that effectively reduces spatial dimensions by averaging values within a given spatial vicinity (pool window). The mathematical definition for a 2D average pooling operation, applied to each channel independently, is given by:

$$y[i,j] = \frac{1}{K_h \times K_w} \sum_{m=0}^{K_h-1} \sum_{n=0}^{K_w-1} x[i+m, j+n] \tag{13}$$

  where: $x[i,j]$ is the input feature map, $K_h$ and $K_w$ are the height and width of the pooling window, respectively, and $y[i,j]$ is the output after pooling. For 3D data, such as volumetric data, the equation extends similarly with an additional dimension.

- Conv3D Transpose (Transposed 3D Convolution): Conv3D Transpose, also known as deconvolution, is a convolution operation that increases the spatial dimensions of the input. The mathematical operation can be understood as the reverse of Conv3D:

$$y[i,j,k] = \sum_{d=0}^{D-1} \sum_{h=0}^{H-1} \sum_{w=0}^{W-1} x'[i-d, j-h, k-w] \cdot w[d,h,w] + b \tag{14}$$

  where: $x'$ is the input feature map, $w$ is the 3D kernel (filter), $b$ is the bias term, and $y[i,j,k]$ is the output at position $(i,j,k)$.

# Figures

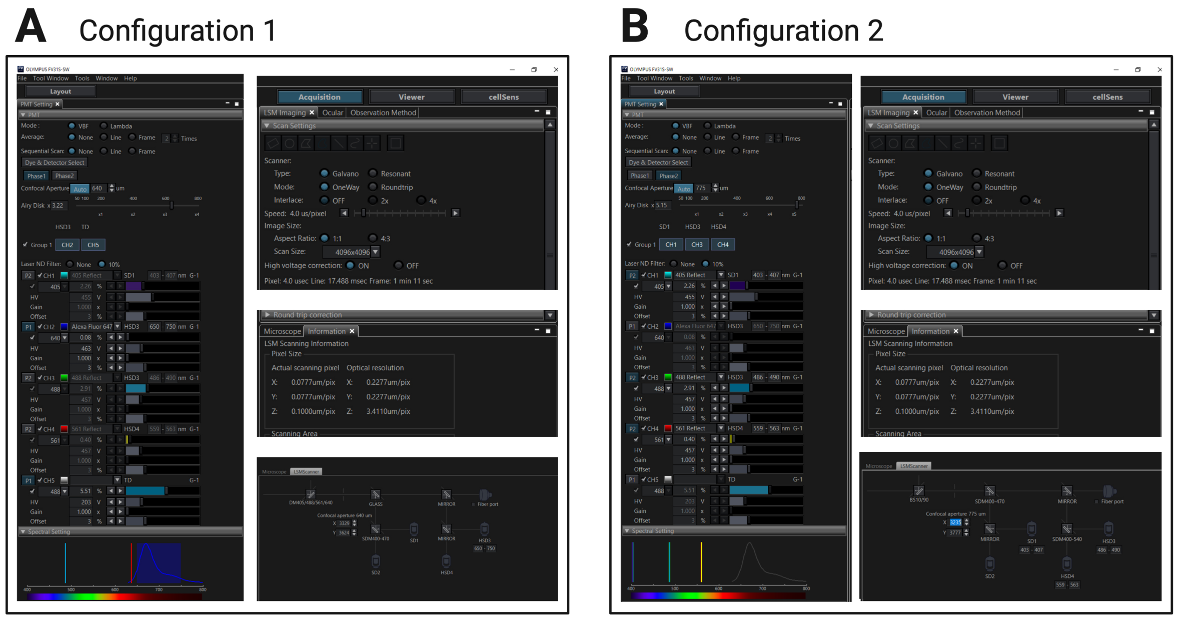


Figure S1: Fluoview Settings: (A): Screenshots of fluoview software showing settings for configuration 1 (labeled as Phase 1 in the software). (B): Screenshots of fluoview software of configuration 2 (Phase 2).

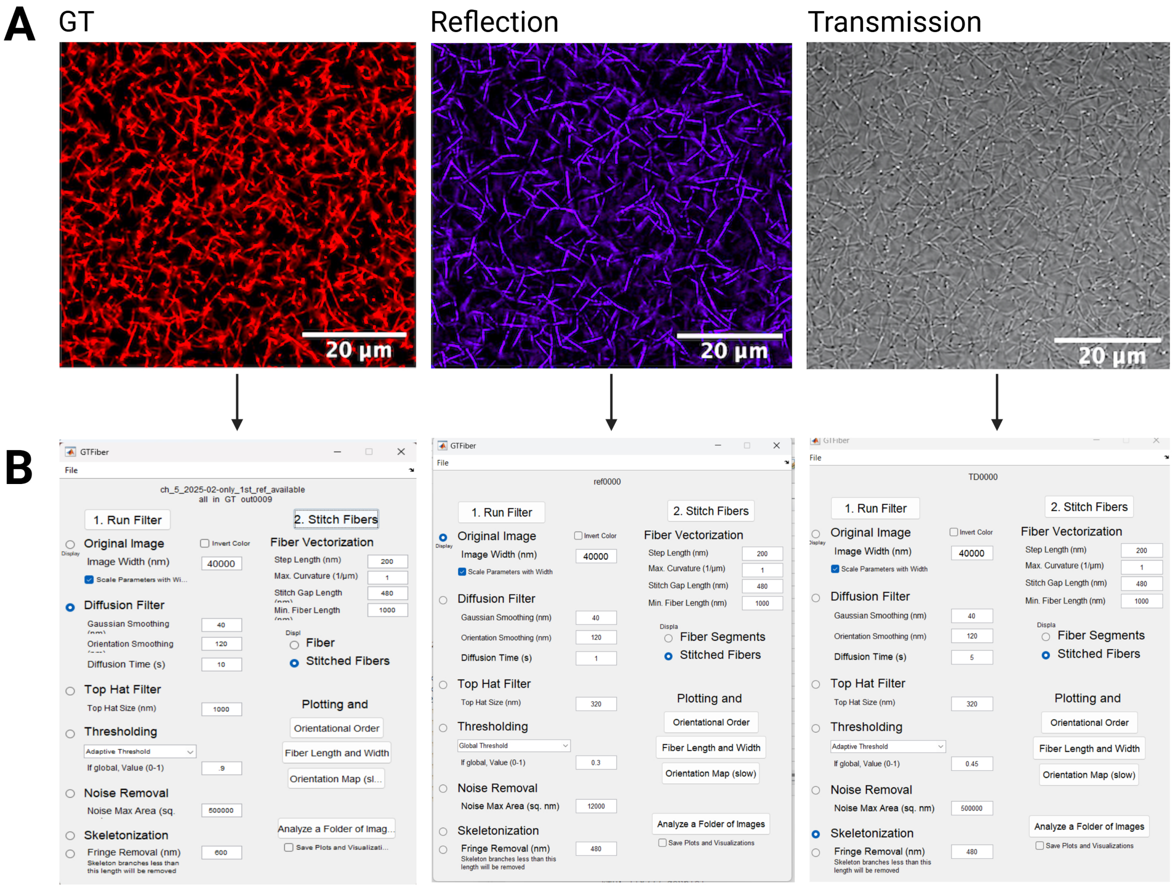


Figure S2: Comparison of GT to denoised GT: (A): GT and denoised GT images acquired approximately 10$\mu$m above the glass bottom of the Petri dish. Intensity profiles along the dashed white line on the images. (B): GT and Denoised GT images acquired at a depth of approximately 50$\mu$m above the glass bottom of the Petri dish.

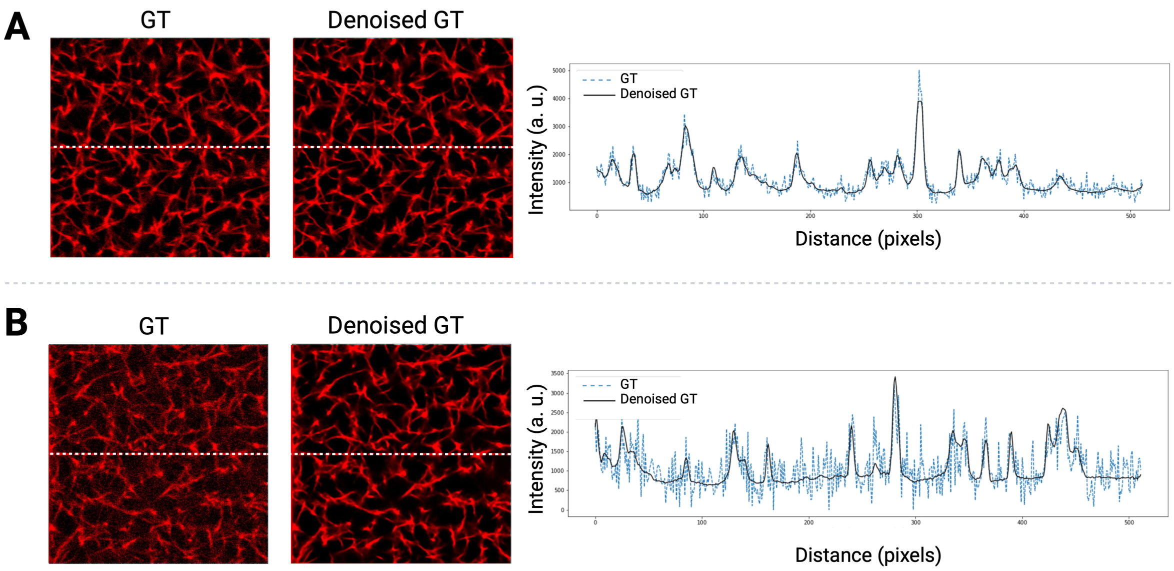


Figure S3: GTFiber Settings: (A): GT, RCM (at 405 nm), and transmission images of the same focal plane within a fibrin scaffold. (B): GTFiber screenshots showing settings for each image type.

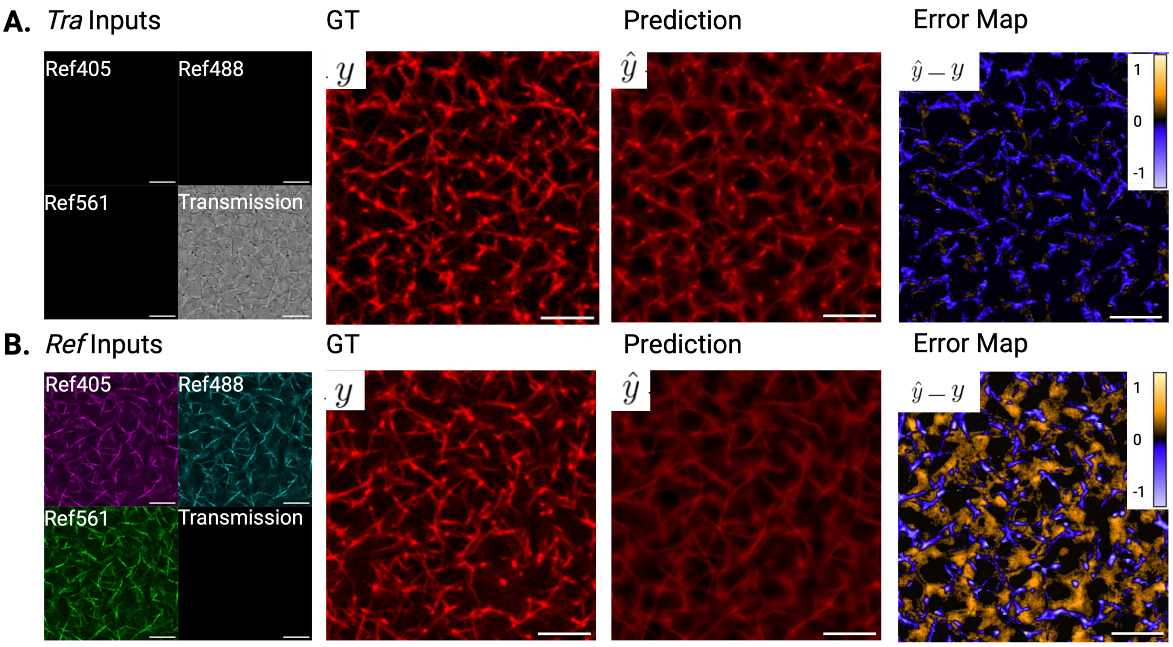


Figure S4: Performance of $Tra$ (A), and $Ref$ (B). The error map image ($\hat{y} - y$) is computed from GT ($y$) and prediction ($\hat{y}$)

.

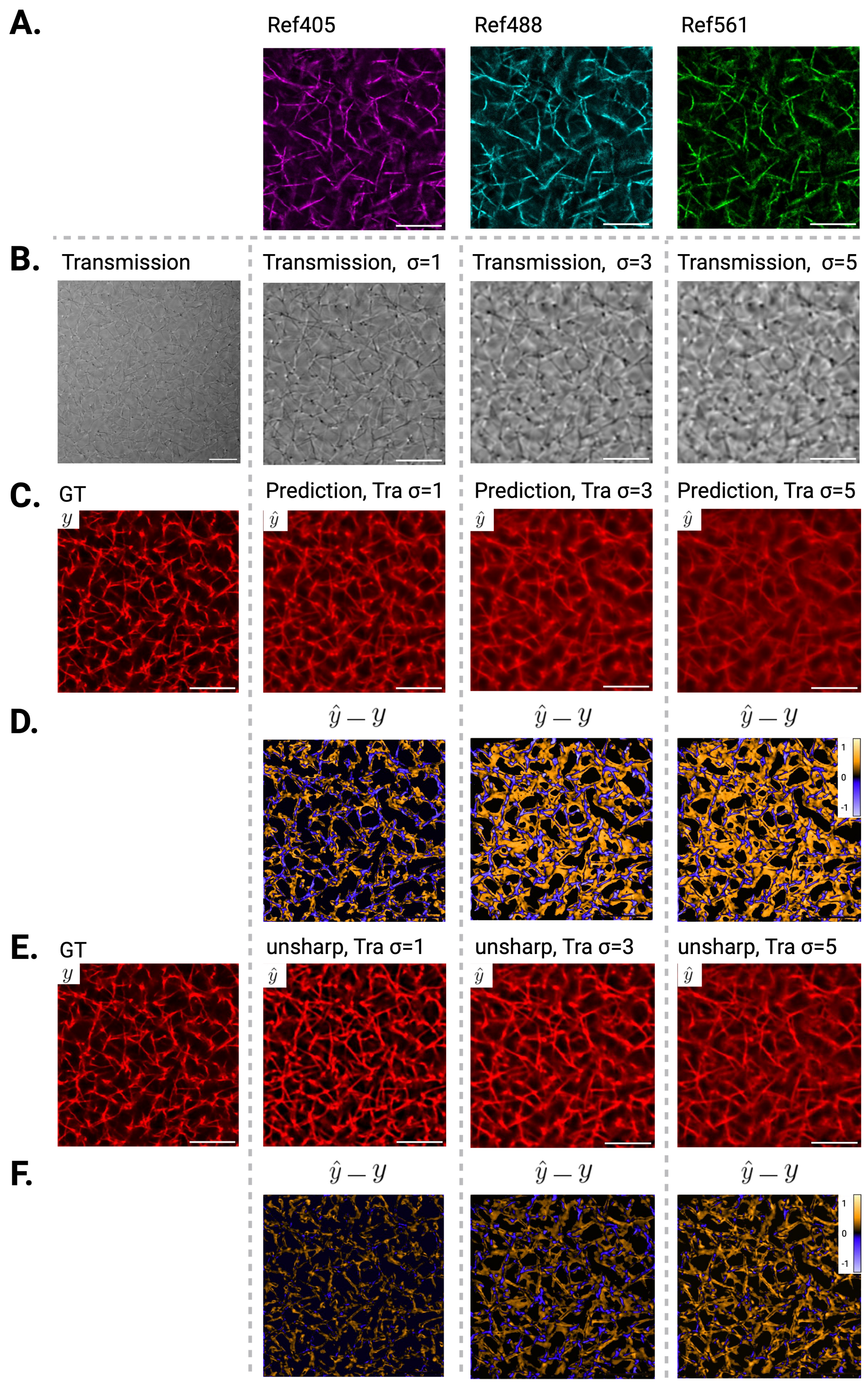


Figure S5: Effect of a blurred transmission channel: (A): RCM inputs for a specific ROI. (B): Transmission image of the same ROI blurred using a Gaussian filter of $\sigma = 1$, 3, or 5.(C): GT along with predictions trained and tested using the different blurred transmission models. (D) Error maps of each model. (E): Prediction in (C) with an applied sharpening mask. (F): Error maps of predictions presented in (E).

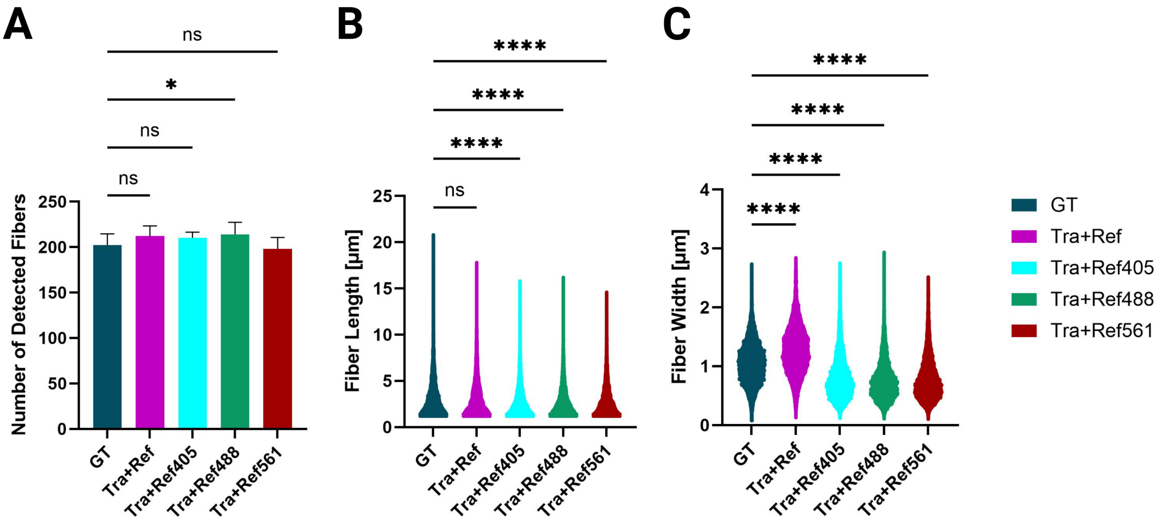


Figure S6: RCM wavelength Contributions: Distributions per slice of number of detected fibers (A), fiber length (B), and fiber width (C) for GT, $Tra + Ref$, $Tra + Ref405$, $Tra + Ref488$ and $Tra + Ref561$ predictions.

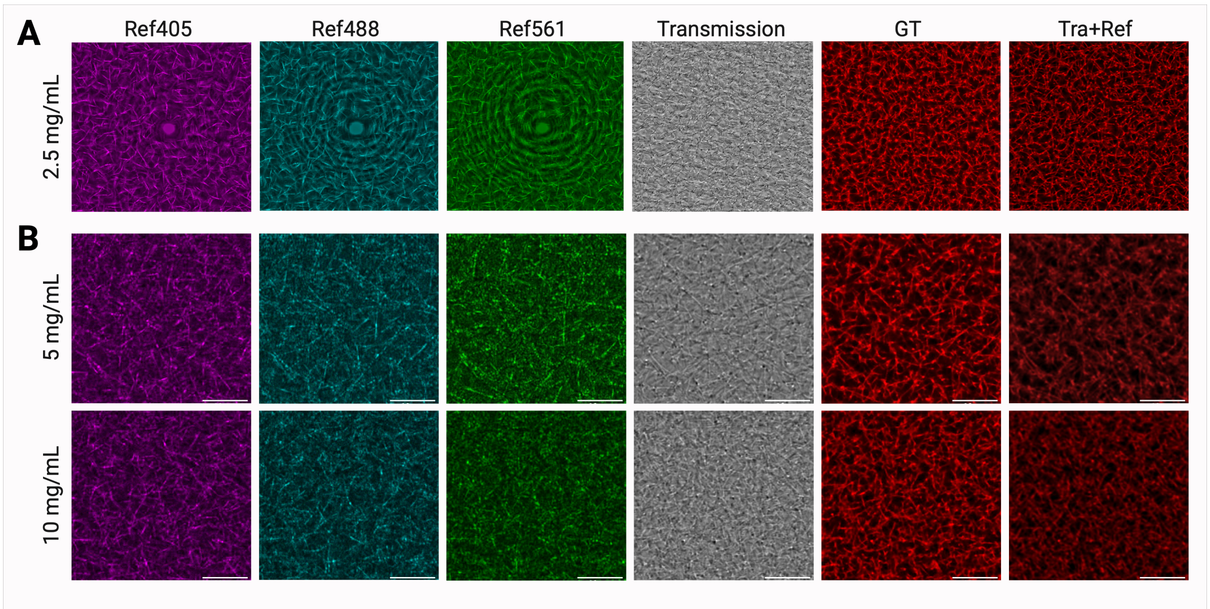


Figure S7: Generalizability of $Tra + Ref$: (A) Example of inputs, RCM (405/488/561) nm and transmission, along with GT and prediction of a 2.5 mg/mL fibrin scaffold around RCM interference ring artifacts. (B) Examples of inputs, RCM (405/488/561) nm and transmission, along with GT and prediction of higher density samples at 5 mg/mL and 10 mg/mL. Scale bars: $10\mu$m.
